# Study on the distribution of ixodid ticks of cattle in pastoral areas of Yabello district, Borana zone, Oromia, Ethiopia

**DOI:** 10.1016/j.parepi.2021.e00200

**Published:** 2021-01-16

**Authors:** Minwyelet Ayana, Abaynew Gelaye, Haben Fesseha, Mesfin Mathewos

**Affiliations:** a Guagusa shikudad District Veterinary Clinic, Awi zone, Amhara region, Ethiopia; b Guangua District Veterinary Clinic, Awi Zone, Amhara Region, Ethiopia; c Wolaita Sodo University, School of Veterinary Medicine, Veterinary Surgery and Diagnostic Imaging, P.O. Box 138, Wolaita Sodo, Ethiopia; d Wolaita Sodo University, School of Veterinary Medicine, Veterinary Pathology, P.O. Box 138, Wolaita Sodo, Ethiopia

**Keywords:** Amblyomma, Boophilus, Hyalomma, Rhipicephalus, Risk factors, Tick, Yabello district, FAO, Food and agricultural organization, TBD, Tick borne disease

## Abstract

**Background:**

Ticks and tick-borne diseases cause major losses in the livestock economy. From both human and veterinary viewpoints, ticks are very important vectors.

**Methods and results:**

This cross-sectional research was conducted to study the prevalence, tick distribution, and related risk factors in the Borana pastoral region of the Yabello district, Oromia regional state, Ethiopia. A total of 445 cattle were examined for the presence of tick infestation collected from different body parts of the cattle and 89.89% (400/445) of indigenous cattle harbor a total of 7,778 adult ticks belonging to four different genera of ticks namely *Rhipicephalus* (78.31%), *Amblyomma* (13.46%), *Boophilus* (7.01%), and *Hyalomma* (1.22%). Besides, *Rhipicephalus pulchellus* (73.17%) and *Amblyomma varigatum* (7.57%) were among the most prevalent tick species identified whereas *Hyalomma dromedari* (0.08%) was found to be the least one. In this study, high proportions of ticks were collected from the head and ear (34.57%) followed by anus and vulva (29.47%), scrotum/udder (19.18%), dewlap and neck (8.77%), brisket (7.16%) and belly and back (0.85%). Moreover, a high proportion of *Amblyomma* species were collected from scrotum/udder (47.76%) and brisket (38.01%); *Rhipicephalus* species from head and ear (41.14%), anus and vulva (33.64%); *Hyalomma* species from scrotum/udder (41.05%), anus and vulva (28.42%) and dewlap and neck (20%) whereas most *Boophilus* species were collected from anus and vulva region (32.48%) and head and ear (31.19%). A statistically significant difference (*P* < 0.05) was observed among potential risk factors like age group being higher in adult animals and different kebeles such as Dharito kebele and Dida Tuyoura ranch.

**Conclusion:**

The result of this survey indicates that economically important ticks are widespread throughout the study areas and their presence in abundance is alerting. To minimize losses attributed to ticks and tick-borne disease cost-effective control strategy should be designed.

## 1 Introduction

Livestock and livestock products play a significant role in Ethiopia's socio-economic growth. In addition, livestock helps rural agricultural households as a source of security and additional cash income. Draft animals provide strength for many peasant agricultural holdings to be cultivated. The effects of ticks and tick-borne diseases (TBD) that impair the quality of hides and skins are at risk for cattle and have resulted in a huge economic loss in foreign export earnings. It also contributes to reduced growth rates, reduced milk production, and also to death (Tiki and Addis, 2011; Wolde and Mohamed, 2014).

Ticks are obligatory, blood-feeding ectoparasites of vertebrates (especially mammals and birds) belonging to the Arachnida class, order Acari (Shichibi et al., 2017; Shane et al., 2017). When ticks are attached to a host for a blood meal, various effects can result in severe losses due to tick concern, loss of blood, damage to hides and skins, and the introduction of toxins (Meaza et al., 2014). They are somewhat similar to mites, but they are larger and all feed only as parasites. There are two main types of ticks in families. Argasidae or Argasides as well as Ixodidae or Ixodides. Argasid ticks are often called soft ticks because their bodies do not have hard plates (Moges et al., 2012; Walker, 2003).

Ticks are very effective vectors of both human and veterinary diseases. Through their strong mouthpieces, ticks damage the skin of farm animals, leading to a decline in the quality of the leather and as a result, to substantial economic losses. In addition, every tick bite facilitates secondary infections, particularly in the tropics (bacteriosis, mycosis, maggot infestations), and this worsens the damage (Brossard, 1998; Desalegn et al., 2015; Ali and de Castro, 1993).

*Amblyomma*, *Boophilus*, *Haemaphysalis*, *Hyalomma*, and *Rhipicephalus* are the main tick genera found in Ethiopia (Shane et al., 2017; Robbins, 2012). The most important and widespread tick species are *A. variegatum* (vector of *Cowdria ruminantum* and *Theileria mutans*) and *Boophilus decoloratus* (vector of *Anaplasma marginale* and *Babesia bigemina*). There is no report on the presence of *R. appendiculatus* (vector to *T. parva*). The effects of ticks have been shown to be low on indigenous cattle relative to exotic breeds. However, there are more than 50 species known to occur in the world (Shichibi et al., 2017; Tamerat et al., 2015; Mekonnen et al., 2001; Troncy et al., 1989).

The major tick genera reported in southwestern Ethiopia during the 1989–1991 tick distribution survey were *Amblyomma* (40%), *Rhipicephalus* (37%), *Boophilus* (21%), *Hyalomma* (1.5%), and *Haemaphysalis* (0.5%). *Amblyomma*, *Rhipicephalus*, and *Boophilus* ticks are primarily livestock parasites. The remaining species occur in limited numbers and have little practical significance to livestock production in the region. *A. cohaerens* and *B. decoloratus* predominates in southwestern Ethiopia, these two species constitute more than 40% of the total collections. Tick population levels are usually low in local cattle most of the time of year and the number rises during the rainy season (Mekonnen et al., 2001).

Ticks have a direct and indirect impact on hosts. Heavy tick infestation produces a tangible effect generally known as tick worry which results in significant blood loss, reduced productivity, reduced weight gain, local skin infection, restlessness, and lesions predispose the animal to myiasis and also cause tick paralysis (Wall and Shearer, 2001). The main bovine tick-borne diseases in Ethiopia include anaplasmosis, babesiosis, cowdriosis, and theileriosis (Moges et al., 2012). Nowadays, the direct losses caused by tick infestations tend to be more economically important than the losses caused by tick-borne diseases. This is mainly due to the fact that the most important tick-borne diseases, East Coast Fever and acute theileriosis caused by *Theileria parva* and *Theileria annulata*, and their vectors have not yet been reported in Ethiopia. However, the impact of ticks on improving the production of indigenous stocks cannot be ignored and long-term control strategies should be developed (Fesseha and Mathewos, 2020; Feleke, 2003).

The direct application of acaricides to host animals is the most widely used method for the efficient control of ticks. Acaricides, however, are costly and can harm the environment: their use should be minimized and incorporated into alternative approaches. Farmers need to understand the biology and ecology of the tick species on their land in order to optimize the use of acaricides since an effective control strategy should use the available compounds at the most appropriate application frequency (Minjauw and Mcleod, 2003; Mideksa et al., 2017).

Ticks are prevalent in all agro-ecological areas in Ethiopia In Ethiopia, ticks are common in all agro-ecological zones (Shichibi et al., 2017; Bayew and Ewnetu, 2019; Misgana, 2017) and the major genera reported are *Amblyomma*, *Boophilus*, *Haemaphysalis*, *Hyalomma*, and *Rhipicephalus* (Moges et al., 2012; Mekonnen et al., 2001; Bedasso et al., 2014; De Castro, 1994). Ethiopia's environmental situation and vegetation are conducive to ticks. Up-to-date information on the importance of ticks in different ecological zones is therefore essential. The objective of this study was therefore to identify the major tick species in the area and their associated risk factors.

## 2 Methods

### 2.1 Study area

**Fig. 1:**
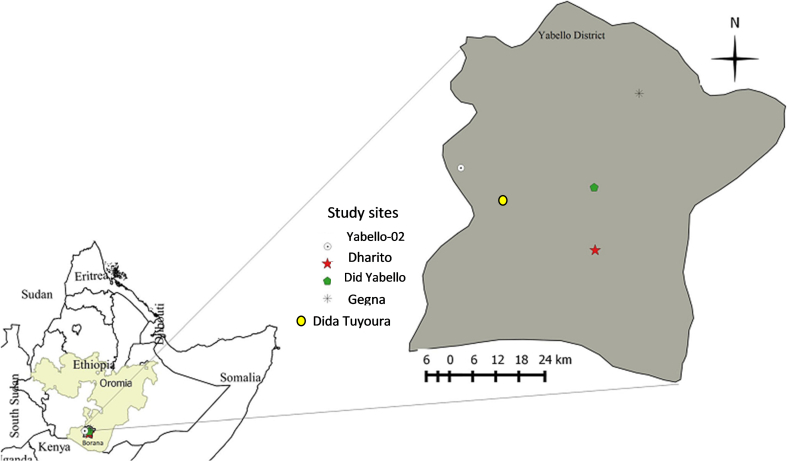
The map of the study area (four villages in Yabello District, Borana Zone, Oromia Regional State, southern Ethiopia) (Note: The boundaries are unofficial).

The study was carried out between October 2013 and March 2014 in the Yabello district of the Borana area of the regional state of Oromia, approximately 570 km from Addis Ababa. In the Borana area, the livestock population is estimated to be 1,496,652 cattle, 452,177 goats, 173,021 sheep, 106, 366 camels, 13,945 mules and 61,699 donkeys. The zone has a semi-arid climate for the most part. With minor seasonal fluctuations and rainfall varying from 350 mm to 900 mm, the annual temperature varies between 21 °C and 38 °C, with substantial spatial and temporal variability in quantity and distribution (CARE-ETHIOPIA: Value chain analysis of milk and milk products in Borana pastoralist area, 2009) (See Fig. 1).

### 2.2 Study animals

All indigenous zebu cattle herds raised under an extensive management scheme, of both sexes and various age groups, are included in this study. The study animals were classified as young or adult based on dentition. Those animals without erupted permanent incisor teeth were classified as young, while those with one or more pairs of erupted permanent incisor teeth were classified as an adult (Wakeman and Pace, 1983).

### 2.3 Study design

From October 2017 to March 2018, a cross-sectional study was conducted on local indigenous cattle (Boran breed) of the Yabello district to classify the main tick species, their predilection locations, and their related risk factors.

### 2.4 Sample size determination and sampling method

The sample animals were selected by systematic random sampling techniques. The required sample size for the analysis was calculated by Thrusfield's formula, (Thrusfield, 2018) using an estimated prevalence of 50% and a precision level of 5%.

$$n=\frac{1.96^{2}p\;\exp\left(1-p_{\exp}\right)}{d^{2}}$$

where; n = required sample size, P_exp_ = expected prevalence, d = desired level of precision at 95% confidence interval.

Therefore, the required sample size was 384, but a total of 455 cattle were examined for the presence of ixodid ticks.

### 2.5 Tick collection, preservation, and identification

Once the selected animals were restrained, all visible adult ticks were collected from the surface of the body of the cattle. Ticks were carefully and gently extracted in a horizontal pull to the surface of the body. The ticks collected were kept in universal bottles containing 70% ethyl alcohol and labeled with the date of collection, predilection sites, age, and sex of the hosts. Then the specimens were transported for counting and labeling to the Yabello veterinary laboratory. The ticks were counted and then classified using a stereomicroscope according to standard identification keys given by (Walker et al., 2003) to the level of genera and species.

### 2.6 Data management and statistical analysis

Data were entered and recorded in a Microsoft Excel sheet and analyzed for descriptive statistics using STATA version 13. The association between tick prevalence and potential risk factors was based on a Chi-square test. Where the *p*-value is less than 0.05, a statistically significant association between variables was considered.

## 3 Results

### 3.1 Tick infestation in cattle and potential risk factors

In the current study, 400 of the 445 cattle tested for tick infestation and were found to be positive for tick infestation giving an overall prevalence of 89.89%. Of these animals, a total of 7778 adult ixodid ticks have been collected. According to the present study, four different tick genera were infested with both sexes of animals, with a prevalence rate of 90.12% (172/192) for females and 89.58% (228/253) for males, respectively.

The highest infestation rate was observed in adult cattle 93.27% (277/297) followed by young 83.11% (123/148). Regarding the study sites, higher tick infestation was recorded in Dharito (100%), Dida Tuyoura ranch (100%), followed by Yabello-02 (89.86%), Gegna (84.62%), and Dida Yabello (83.19%). Moreover, the highest prevalence of tick infestation was recorded in February (100%), December (90.67%), and January (87.70%). Risk factors such as age, sex, month, and kebele were considered in this study and there was significant variation (*P* < 0.05) in prevalence was observed. However, there was no significant difference in prevalence between female and male animals (*P* > 0.05) (Table 1).

**Table 1:** Prevalence of tick infestation and its associated risk factors in Yabello district, 2017/18.

Risk factors	Category	No. examined	No. positive	Prevalence (%)	χ^2^	P-value
Age	Young	148	123	83.11	11.2128	0.001
	Adult	297	277	93.27		
Sex	Female	253	228	90.12	0.0344	0.853
	Male	192	172	89.58		
Month	December	75	68	90.67	8.5361	0.014
	January	309	271	87.70		
	February	61	61	100.00		
Kebeles/ Villages	Dharito	79	79	100.00	25.1949	0.0001
	Dida Tuyoura ranch	61	61	100.00		
	Dida Yabello	119	99	83.19		
	Gegna	117	99	84.62		
	Yabello-02	69	62	89.86		
Total		445	400	89.89		

### 3.2 Tick species identification and their burden

In the present study, ten species of ixodid ticks belonging to four genera were identified in the district. The tick genera identified in this study were: *Rhipicephalus* (78.31%), *Amblyomma* (13.46%), *Boophilus* (7.01%), and *Hyalomma* (1.22%). The most abundant tick species in the district was found to be *Rh. pulchellus* accounting for 73.17% of the total collection followed by *A. variegatum* (7.57%) (See Table 2).

**Table 2:** Identified tick species in different sites of Yabello district, 2017/18.

Tick	Study sites					Total	Proportion (%)
	Dharito	Yabello 02	Gegna	Dida Yabello	Did Tuyoura		
***Amblyomma***	**189**	**114**	**329**	**233**	**182**	**1047**	**13.46**
*A. variegatum*	59	65	179	169	117	589	7.57
*A. gemma*	123	44	147	63	65	442	5.68
*A. lepidium*	7	5	3	1	0	16	0.21
***Rhipicephalus***	**1170**	**470**	**1464**	**1291**	**1696**	**6091**	**78.31**
*Rh. pulchellus*	1144	404	1348	1195	1600	5691	73.17
*Rh. e. evertsi*	12	57	68	56	68	261	3.36
*Rh. pravus*	14	9	48	40	28	139	1.79
***Hyalomma***	**28**	**9**	**31**	**24**	**3**	**95**	**1.22**
*H. truncatum*	23	3	21	21	0	68	0.87
*H. dromedari*	2	2	1	1	0	6	0.08
*H. m. rufipes*	3	4	9	2	3	21	0.27
***Boophilus***	**105**	**114**	**65**	**147**	**114**	**545**	**7.01**
*B. decoloratus*	105	114	65	147	114	545	7.01
**Total**	**1492**	**707**	**1889**	**1695**	**1995**	**7778**	**100.00**

Out of the total ticks collected, sex determination and enumeration was performed. The finding revealed that 5289 were males while the rest of 2489 were females with an overall male to female ratio of 2.1:1. *Rhipicephalus* species were the most commonly identified followed by *Amblyomma, Boophilus*, and *Hyalomma* (see Table 3)

**Table 3:** Count of tick species by sex and M: F sex ratio, Yabello district, 2017/18.

**Tick species**	**Male**	**Female**	**Male to female (M: F) ratio**
***Amblyomma***	**932**	**115**	**8.1:1**
*A. variegatum*	549	40	13.7:1
*A. gemma*	370	72	5.1:1
*A. lepidium*	13	3	4.3:1
***Rhipicephalus***	**4239**	**1852**	**2.3:1**
*Rh. pulchellus*	3983	1708	2.3:1
*Rh. e. evertsi*	165	96	1.7:1
*Rh. pravus*	91	48	1.9:1
***Hyalomma***	**53**	**42**	**1.3:1**
*H. truncatum*	35	33	1.1:1
*H. dromedari*	5	1	5.:1
*H. m. rufipes*	13	8	1.6:1
***Boophilus***	**65**	**480**	**0.1:1**
*B. decoloratus*	65	480	0.1:1
**Total**	**5289**	**2489**	**2.1:1**

### 3.3 Body distribution of tick species

Ticks were collected from different body regions of the animal and high proportions of ticks were collected from the head and ear (34.57%) followed by anus and vulva (29.27%), scrotum/ udder (19.38%), dewlap, and neck (8.77%), brisket (7.16%) and belly and back (0.86%). In this study, a high proportion of *Amblyomma* species were collected from scrotum/udder (48.59%) and brisket (37.41%). *Rhipicephalus* species were collected mostly from head and ear (41.24%), anus, and vulva (33.48%). *Hyalomma* species were abundantly collected from scrotum/udder (39.78%), anus and vulva (29.03%), and dewlap and neck (20.43%) whereas, 32.48% and 31.19% of *Boophilus* species were collected from anus and vulva region and head and ear, respectively (see Table 4:).

**Table 4:** Proportion and body distribution of identified tick species in Yabello district, 2017/18.

**Body region**	***Amblyomma***		***Rhipicephalus***		***Hyalomma***		***Boophilus***		**Total**	**Proportion (%)**
	**No.**	**%**	**No.**	**%**	**No.**	**%**	**No.**	**%**		
Head and ear	10	0.96	2506	41.14	3	3.16	170	31.19	2689	34.57
Dewlap and neck	81	7.74	520	8.54	19	20.00	62	11.38	682	8.77
Brisket	398	38.01	128	2.10	7	7.37	24	4.40	557	7.16
Belly and back	19	1.81	41	0.67	0	0.00	6	1.10	66	0.85
Scrotum/udder	500	47.76	847	13.91	39	41.05	106	19.45	1492	19.18
Anus and vulva	39	3.72	2049	33.64	27	28.42	177	32.48	2292	29.47
**Total**	**1047**	**100.00**	**6091**	**100.00**	**95**	**100.00**	**545**	**100.00**	**7778**	**100.00**

## 4 Discussion

In the present study out of 445 cattle examined in the study period, 400 animals were found to be infested with tick giving an overall prevalence of 89.89%. This finding is in agreement with previous studies of (Abera et al., 2010) who reported 97.8% in cattle from Bedelle district, Southwestern Ethiopia, (Meaza et al., 2014) 211 (91.7%) in Bahir Dar extensive farms, (Shichibi et al., 2017) in Saylem 502 (88.54%), Gesha 183 (91.50%) and Masha 149 (78.84%) in districts of Southern Ethiopia, (Kemal et al., 2016) 2024 (75.7%) in Arbegona District, Southern Ethiopia, (Teshome et al., 2016) 270 (70.31%) in Bishoftu town, (Wogayehu et al., 2016) 68.12% in the high land of Decha woreda. (Tamerat et al., 2015) 82% in Bedelle district, Oromia Regional State, (Alemu et al., 2014) 81.25% in North-West Ethiopia, and (Wolde and Mohamed, 2014) 65.5% in Sodo zuriya districts, Wolaita Zone, (De Castro, 1994) where it was stated that more than 80% of the cattle studied were ticks-infested.

In contrast to the current study, lower prevalence of ixodid ticks were reported from (Fesseha and Mathewos, 2020) 42.2% in Hosana district, Hadiya zone, (Tiki and Addis, 2011) with 25.64% tick infestation prevalence in Holeta district and (Zelalem et al., 2016) 146 (38%) in the Chiro district. The variation in the prevalence rate in the study area might be attributed to the agroecological, animal health practice, the management system within their respective study area, level of awareness of farmers about the economic importance of tick and tick control strategy.

The age-wise tick prevalence of the current study showed that there was a higher infestation rate was recorded in adult animals 93.27% (277/297) than young age groups 83.11% (123/148) are probably associated with larger body size and tick have more surface for blood-feeding. Thus, hard ticks attach to the host for a long time, so older animals are more likely to have ticks. Moreover, as age increases the immunity of the body starts to decline and this is true in the case of old animals. Similarly, this finding was in agreement with the previous work of (Shichibi et al., 2017) with prevalence higher in adult (93.97%) than young (78.71%), (Meaza et al., 2014) with high prevalence with increasing age interval adult (62.7%) and young (85.1%), (Ayana et al., *2013*) and (Okello-Onen et al., 1999). This was not consistent with the previous report of (Fesseha and Mathewos, 2020) where a higher tick prevalence 28% (77/275) was recorded in adult one than other age groups, (Kemal et al., 2016) also reported a prevalence of 98.4% in Arbegona District, Southern Ethiopia, (Yalew et al., 2017) with the highest affected young age cattle 56 (43.41%) than other age groups. There might be due to different reasons. Young cattle were managed in the house until they become stronger so that the chance of acquiring the tick was less than the adult one which was kept in an outdoor system. Besides, there was variation in age, there was a significant association (*p* < 0.05) with the prevalence of tick.

During the study period out of 7778 ticks collected from cattle the genera *Rhipicephalus*, *Amblyomma*, *Boophilus*, and *Hyalomma* were identified in order of predominance. The predominant tick species during the study period was *Rh. pulchellus* accounting for 73.17% of the total collection. In Ethiopia, *Rh. pulchellus*, “Zebra tick”, is the most predominant ticks inhabiting dry, semi-arid, and bushland and are found to be most abundant with marked seasonal changes. This finding was consistent with the earlier studies by (Ayana et al., 2013) in Borana pastoral area, Solomon and Kaaya, (Solomon and Kaaya, 1996) and (Regassa, 2000) in western Ethiopia, (Walker, 2003) in the Rift Valley and eastwards, (Manuri and Tilahun, 1991) in the drier lowlands of Harar and (Seyoum, 2001) at Girana valley in the North Wollo Zone. In contrast, a study by (Fesseha and Mathewos, 2020) also reported four different genera of ticks namely *Hyalomma* (11.9%), *Amblyomma* (10.7%), *Boophilus* (10.2%), and *Rhipicephalus* (9.4%).

In the present study, *Rhipicephalus* was the most dominant tick genera which accounts for 78.31% followed by *Amblyomma* (13.46%), *Boophilus* (7.01%), and *Hyalomma* (1.22%). According to (Wasihun and Doda, 2013) were also stated that three out of four of our tick genera were prevalent in Ethiopia. This was in contrast to the previous report by (Kemal et al., 2016) in Arbegona District, (Wasihun and Doda, 2013) in Humbo district, (Ayalew et al., 2014) in central Oromia, and (Yehualashet et al., 1995) at Haramaya University where *Amblyomma* was found to be the most abundant tick genera. Besides, (Shane et al., 2017) identified *Boophilus* as the dominant tick in Tiyo District. This could be due to the difference in the season during which the study was conducted (De Castro, 1994; Abera et al., 2010).

The sex-wise distribution revealed that the number of male ticks are more dominant than the number of female ticks except for *Boophilus decoloratus* whose males are less than the female numbers and a similar result was reported by (Ayana et al., 2013). In contrast to the current finding, (Fesseha and Mathewos, 2020) reported that females (30.4%) were more prevalent than male (28.6%). This is due to fully engorged female tick drop-off to the ground to lay eggs while males tend to remain permanently attached to the host for up to several months. The increased female to male ratio of *Boophilus decoloratus* might be due to the small size of males which creates the difficulty of finding it during collection. This finding was in line with the previous findings of (Shichibi et al., 2017) 351 (89.77%) in Saylem, Gesha, and Masha Districts, (Zelalem et al., 2016) 70 (41.9%) in Chiro district and (Belayneh and Bogale, 2016) 169 (78.6%) in Jabitehnan Woreda.

In the present study, high proportions of ticks were collected from Head and ear (34.57%), followed by Anus and vulva (29.47%), Scrotum/udder (19.18%), Dewlap and neck (8.77%), Brisket (7.16%), Belly and back (0.85%). Moreover, a high proportion of *Amblyomma* species were collected from scrotum/udder and brisket, *Rhipicephalus* species from head and ear and anus and vulva, *Hyalomma* species from scrotum/udder, anus and vulva and dewlap and neck and *Boophilus* species from anus and vulva region and head and ear.

## 5 Conclusion

In conclusion, ten tick species belonging to genera of *Amblyomma*, *Rhipicephalus*, *Boophilus,* and *Hyalomma* were identified. *Rhipicephalus pulchellus* and *A*. *varigatum* were the most abundantly distributed species on cattle of Borana pastoral area at Yabello. The age and sex of cattle were found to be an important determinant for tick population dynamics. Irrespective of the seasons, a high total tick count was found on adult cattle than the youngest; and on females than males.

It is well known that ticks cause severe economic losses either by transmitting a variety of diseases or by their evident damage to hides and skins. The result of this survey indicates that economically important ticks are widespread throughout the study areas and their presence in abundance is alerting. Therefore, to minimize losses attributed to ticks and tick-borne disease cost-effective control strategy should be designed.

## Data availability statement

The data will be provided upon the request of the corresponding author.

## Funding statement

The current study was conducted without the support of funding sources.

## Authors' contributions

MA, AG, HF, and MM contributed to data gathering and manuscript write-up, HF was involved in data analysis and write-up as well as editing of the manuscript. All authors have approved the submission of the manuscript.

## Consent for publication

Not applicable.

## Declaration of Competing Interest

All authors declared no competing conflict of interest.
